# An electron microscopic and biochemical study of the potential protective effect of ginger against Cadmium-induced testicular pathology in rats

**DOI:** 10.3389/fphys.2022.996020

**Published:** 2022-10-03

**Authors:** Moustafa E. Motawee, Ahmed A. Damanhory, Hany Sakr, Mohamed Mansour Khalifa, Tarek Atia, Mohamed M. Elfiky, Muhammad Maher, Hader I. Sakr

**Affiliations:** ^1^ Department of Histology and Cytology, Faculty of Medicine, Al-Azhar University, Cairo, Egypt; ^2^ Medicine Program, Batterjee Medical College, Jeddah, Saudi Arabia; ^3^ Department of Biochemistry, Faculty of Medicine, Al-Azhar University, Cairo, Egypt; ^4^ Department of Pathology and Laboratory Medicine, VAMC, Northeast Ohio Health Care System, Louis Stokes, Cleveland, OH, United States; ^5^ Department of Medical Physiology, Faculty of Medicine, Cairo University, Cairo, Egypt; ^6^ Department of Medical Physiology, College of Medicine, King Saud University, Riyadh, Saudi Arabia; ^7^ Department of Medical Laboratory Sciences, College of Applied Medical Sciences, Prince Sattam Bin Abdulaziz University, Al-Kharj, Saudi Arabia; ^8^ Department of Anatomy and Embryology, Faculty of Medicine, Menoufia University, Menoufia, Egypt

**Keywords:** cadmium, ginger (*Zingiber officinale*), testicular tissue, reduced glutathione (GSH), malondialdehyde (MDA), nitric oxide (NO)

## Abstract

**Background:** Cadmium (Cd) is a toxic heavy metal used in many industries. Since the second half of the 20th century, legislation on Cd use was put to limit the exponential rise in its environmental levels. This study aimed to investigate Cd’s functional and ultrastructural changes on rats’ reproductive systems and the role of Zingiber officinale (Ginger) in protecting against Cd-induced toxicity.

**Methods:** Thirty adult male albino rats were randomly assigned into three equal groups (*n* = 10); control, Cd-exposed/untreated, and Cd-exposed/Gin-treated. Rat testes were weighed, and testicular tissue sections were examined under the electron microscope. Semen analysis, morphological examination of spermatozoa, and serum levels of luteinizing hormone (LH), follicle-stimulating hormone (FSH), and testosterone were measured. In addition, testicular tissue homogenates were analyzed for malondialdehyde (MDA), nitric oxide (NO), and reduced glutathione (GSH) levels.

**Results:** Cd-induced significant reduction in the mean testicular weight and GSH levels and plasma testosterone, LH and FSH levels with a concomitant increase in testicular MDA and NO levels. There was also a deterioration in semen analysis parameters and spermatozoa morphology, with testicular structural damage in the form of architecture distortion and necrosis of seminiferous tubules and testicular interstitial cells. Daily administration of ginger for 4 weeks protected against CD-induced toxicity, preserving tissue architecture, improved plasma levels of testosterone, LH and FSH and testicular levels of GSH, and reduced testicular levels of MDA, NO.

**Conclusion:** Ginger has a protective effect on Cd-induced deterioration of testicular tissue’s structural and functional integrity by improving testicular tissue antioxidant capacity and steroid production, which ameliorates sex hormone levels in the blood.

## Highlights

- Cadmium has hazardous effects on testicular chemistry and histology by disrupting normal testicular oxidative mechanisms reflected on blood sex hormone levels.
- Ginger orally administered at a dose of 4 mg/kg/day for four weeks reversed most of the Cd-induced adverse effects.

## Introduction

Cadmium (Cd) naturally occurs in the environment as a pollutant derived from agricultural and industrial sources. It is a soft, silver-white, divalent metal; found in Zinc ores. It is a byproduct of smelting, mining, and refining lead and copper ores (Dharmadasa *et al.,* 2017). Cd is used in many industries, including Nickle batteries, cadmium-selective sensors, nuclear reactors, and photocopier drums (Taki, 2013). It is also used to colour glass, stabilize plastic, and create a corrosion-resistant coat in the steel industry. However, the industrial use of Cd is gradually decreasing due to its toxic effects (Rafati Rahimzadeh *et al.,* 2017). A Cd-dependent carbonic anhydrase enzyme was found in marine diatoms; however, Cd has no known biological function in higher organisms (Jensen *et al.,* 2019).

The average daily Cd-exposure level of an average human is 0.15 µg through inhalation and 1 µg from consuming contaminated water and food. Cigarette smoking is another known source of Cd intoxication, as smoking one daily packet deposits 2–4 µg of Cd within lung parenchyma (Prokopowicz *et al.,* 2019). Some of the Cd-induced toxicity was attributed to its chemical similarity to zinc. Cd is poorly metabolized in human bodies. It is filtered by kidneys and gets reabsorbed in the proximal renal tubules with subsequent deposition in various body tissues and organs. By disrupting standard cellular oxidative mechanisms, Cd increases the risk for cancer, cardiovascular diseases, chronic renal diseases, reproductive system dysfunctions, and osteoporosis (Wallin *et al.,* 2013).

The exact mechanisms by which Cd induces its toxic effects on various body organs are still undergoing investigation (Andjelkovic *et al.,* 2019). The spermatogenic cells are among the most rapidly dividing cells in the body. They are vulnerable to various types of injury, including heavy metal intoxication. Being very prone to the deleterious effects of heavy metals, most testosterone-producing Leydig cells in testes and steroid-hormone-producing cells in adrenal glands usually become suppressed when exposed to such heavy metals, e.g., mercury, Cd, cobalt, and aluminium (Rana, 2014).

Heavy metal chelation effectively prevents metal-induced toxicity by enhancing the mobilization and excretion of metallic cations. Nutrients affect bioavailability, toxico-dynamics and transport of heavy metals to target organs and influence the immunologic, biochemical, or cytologic functional responses to heavy metals intoxication (Nwokocha *et al.,* 2011). Several studies have investigated the protective effects of nutritional supplements and vitamins against heavy metal-induced testicular injury (Mouro *et al.,* 2019). Nevertheless, increasing evidence suggests that diet and/or metabolic differences may influence heavy metal uptake and/or excretion (Canuel *et al.,* 2006).

Spices are essential components of the human diet. Various phytochemicals in spices were shown to promote human health and have a protective role in many chronic diseases (Siruguri and Bhat, 2015). Zingiber officinale (Ginger) is one of the most commonly used spices worldwide (Ali *et al.,* 2008). It is widely used in folk medicine due to its known health benefits in various diseases (Banihani, 2018) such as cardiovascular disease (Liu *et al.,* 2013), diabetes mellitus (Hroob *et al.,* 2018), ulcer (Liu D. et al., 2015), Alzheimer’s, depression (Kukula-Koch *et al.,* 2018) and cancer (Chen *et al.,* 2018). The plant form, rhizome, contains several bioactive substances, including volatile oils and nonvolatile pungent compounds, e.g., oleoresin, gingerols, and their dehydrogenated products (Marx *et al.,* 2017). It also contains a host of compounds, which include acid resins, vitamin C compounds (folic acid, inositol, choline and pantothenic acid), gingerol, sesquiterpene, vitamins B3 and B6, volatile oils, and bio-trace elements (Ca^+2^, Mg^+2^, P^−3^ and K^+^) (Egwurugwu *et al.,* 2007). Ginger root potentiates testosterone’s biosynthesis through its antioxidant compounds content and enhances the activity of antioxidant enzymes that protect the reproductive organs from oxidative stress and lipid peroxidation (Banihani, 2018).

Several reports document ginger’s protective effects on different body systems. However, the exact biological and cellular mechanisms underlying its protective role are largely unknown. The current study aimed to: (1) investigate Cd-induced functional and ultrastructural changes in spermatogenic cells of adult albino rat testes and (2) elucidate the underlying mechanisms of the prophylactic effect of ginger at cellular/subcellular levels.

## Methods

### Experimental animals

Thirty adult male albino rats at 12 weeks of age and weighing 200 g m (±20 g) were used in the study. Rats were obtained from the Research Unit, They were kept under observation for 15 days before experimentation for adaptation and to exclude any signs of infection. Rats were housed in plastic cages (3 rats/cage), kept at an average temperature of 22 °C (±4 °C) and relative humidity (45 ± 5%) and exposed to alternating 12-h light/dark cycles. Rats were given free water access and provided with a laboratory diet offered *ad libitum* throughout the study period (4 weeks). All methods are reported following ARRIVE guidelines (Percie du Sert *et al.,* 2020). The experimental protocol was adherent to “the Basel Declaration” principles (Basel Declaration, 2020) and the ethical guidelines of the International Council for Laboratory Animal Science (ICLAS) (Ethics and Animal Welfare Committee, 2020) and was approved by the ethical committee of Prince Sattam Bin Abdulaziz University, with the approval No.: MLAB/022/012.

### Animal group design

Rats were randomly divided into three equal groups (n = 10) as follows:

A. Control Group (I): given 0.5 ml/day of sterile normal saline orally by gavage.
B. Cadmium Group (Cd-treated, II): given 4 mg/kg/day of Cd chloride (CdCl_2_) dissolved in sterile normal saline and given orally by gavage (Bu *et al.,* 2011; Rajendar *et al.,* 2011).
C. Gin-treated Cadmium Group (Gin-treated, III): given 4 mg/kg/day of CdCl_2_ combined with 40 mg/kg/day of ginger, dissolved in sterile normal saline and given orally by gavage (Moselhy et al., 2012).

### Reagents

A. Cadmium Chloride: was purchased from Sigma (St. Louis, MO, United States).
B. Ginger: ground dried ginger root powder obtained from Frontier Natural Products (Norway, IA, United States).

### Animal sacrifice and testicular tissue sample collection

By the end of the experiment, rats fasted for 24 hours; heparinized capillary tubes were used to obtain retro-orbital blood samples that were centrifuged at 10,000 rpm for 20 min, and serum was aliquoted and stored at -70 °C.

Rats were then sacrificed by the “CO_2_ euthanasia” method, as previously described (Underwood and Anthony, 2020). Rats’ scrotums were immediately cut open, and testes were irrigated with normal warm saline to identify vascular beds. The testes were perfused with glutaraldehyde 2% and paraformaldehyde 4% for 25–30 min, then excised out of tunicae coverings and weighed. The average weight of both testes was recorded for each rat. Several fragments (2 mm^3^ each) of testicular tissue were collected and stored at -70°C for electron-microcopy slide preparation. The remainders of testicular tissues were homogenized in 5–10 ml cold buffer (50 mM potassium phosphate, 1 mM EDTA at pH 7.5) per Gram tissue and then ultra-centrifuged g for 15 min at 4 °C. The supernatant was carefully removed and stored at -70°C.

### Biochemical assays

i. Plasma Levels of FSH, LH, and Testosterone: Plasma hormone levels were measured using a competitive immunoassay technique, as previously described (Sharma et al., 2011).
ii. Tissue Levels of MDA, GSH, and NO: MDA levels were measured using the thiobarbituric acid reactive substances (TBARS) test (Jardine *et al.,* 2002). In order to measure GSH levels, 5,5 dithiobis 2-nitrobenzoic acid was utilized (Giustarini *et al.,* 2014). NO was measured as previously described by Zhang *et al.* (2014).

### Semen analysis

The caudal section of epididymis was dissected from the testis and cut into a Petri dish containing 1 cc Ham’s F10 medium (pre-heated to 37°C), a supporting spermatozoa culture medium. Petri dishes were incubated at 37°C for 15 min. Then, the cell suspension was used to evaluate sperm parameters (motility, count, viability, and morphology)

The percentage of motility in spermatozoa was calculated, a hemocytometer lam performed sperm counting, and the viability of spermatozoa was evaluated by cell membrane integrity following the criteria provided by the WHO. The assessment of sperm morphology was qualitative and quantitative and included microscopic evaluation for atypical spermatozoa morphology involving any changes involving the head, neck, mid-piece and tail and the diagnosis of abnormal spermatozoa (Horri *et al.,* 2021).

### Electron microscopy

Tissue blocks, 2 mm^3^ each, were fixed in glutaraldehyde (2%) in 0.1 M phosphate buffer (pH 7.4), transferred to 1% osmium tetroxide, dehydrated in ascending alcohol and propylene oxide grades, and finally embedded in Epon. Ultrathin sections (40–50 nm) were cut using a glass knife, stained with 4% uranyl acetate and 2% lead citrate and examined under JEOL 100 S electron microscope (de Souza Predes *et al.,* 2010).

### Light Microscopy

i. Morphologic evaluation of the spermatozoa:

Sperms’ smears were made on clean and grease-free slides and allowed to air-dry overnight. Each sample slides were stained with hematoxylin and eosin (H&E) and examined at 200X and 400X (Soleimanzadeh and Saberivand, 2013).

ii. Histologic examination of rat testes:

Testicular tissues were processed routinely, embedded in paraffin wax, and 3–5 μm thick sections were cut, stained with H&E, and examined at 200X and 400X (Clancy *et al.,* 2018).

### Statistical analysis

Data were coded and entered using the statistical package SPSS version 24, and the results were expressed as mean ± standard deviation (Mean ± SD). Normality of distribution was evaluated by “Shapiro-Wilk’s test.” Analysis of variance (ANOVA) with the “Bonferroni post hoc test” was done to compare quantitative variables between groups. At *p* ≤ 0.05, results were considered statistically significant, and at *p* ≤ 0.01 were considered highly significant (Chan*,* 2003).

## Results

### Testicular Weight

The weight of testes in both the Cd (-57.44%) and Gin-treated (-19.46%) groups showed a high statistically significant (*p* < 0.01) decrease compared to the control group. However, ginger seemed to have some ameliorating effect on testes’ weight in the Gin-treated group compared to the Cd group, as shown in Table 1.

**TABLE 1:** Comparison of the weight of testis, plasma levels of hormones, testicular tissue levels of cytokines and sperm parameters among the study groups.

Groups (*n* = 10)		Control	Cadmium (%)	Cd & Gin treated (%)
Weight of testis (g)		1.68 ± 0.055	0.715 ± 0.092** (−57.44%)	1.352 ± 0.079**^##^ (−19.46%)
Plasma level (pg/ml) of	LH	10.66 ± 1.544	4.07 ± 1.041** (−61.82%)	7.79 ± 1.513*^##^ (−26.92%)
	FSH	13.02 ± 2.863	4.82 ± 1.471** (−62.98%)	9.79 ± 2.607^#^ (−24.81%)
	Testosterone	29.37 ± 6.148	8.78 ± 2.151** (−70.11%)	20.15 ± 5.160*^##^ (−31.39%)
Testicular tissue level (mmol/mg) of	MDA	1.09 ± 0.073	2.11 ± 0.091** (93.58%)	1.47 ± 0.084**^##^ (34.86%)
	NO	12.14 ± 1.29	25.33 ± 2.286** (108.65%)	16.43 ± 1.239**^##^ (%35.34)
	GSH	57.19 ± 5.896	22.68 ± 2.701* (−60.34%)	46.19 ± 5.127*^#^ (−19.23%)
Sperm	Viability (%)	87 ± 2.38	45.33 ± 2.925** (−47.9%)	73.83 ± 3.436**^##^ (−15.14%)
	Motility (%)	81.17 ± 4.67	41.83 ± 2.544** (−48.47%)	65.33 ± 3.543**^##^ (−19.51%)
	Count (10^6^/ml)	8.485 ± 1.16	3.858 ± 0.685** (−54.53%)	6.467 ± 1.294*^##^ (−23.78%)
	Abnormality (%)	9.995 ± 1.625	32.92 ± 3.398** (229.36%)	15.93 ± 2.184**^##^ (59.38%)

Values are presented as mean ± SD.

Statistically significant (*p* ≤ 0.05) compared to corresponding value in (*): Control group and (^#^): Cd group.

Highly statistically significant (*p* ≤ 0.01) compared to corresponding value in (**): Control group and (^##^): Cd group.

## Biochemical Parameters

1) Plasma Levels of LH, FSH, and Testosterone: Our results showed a high statistically significant (*p* < 0.01) decrease in plasma levels of LH (-61.82%), FSH (-62.98%) and testosterone (-70.11%) in the Cd group compared to the control group. The Gin-treated group showed a statistically significantly (*p* < 0.05) reduced plasma levels of LH (-26.92%) and testosterone (-31.39%); however, plasma levels of FSH showed a statistically insignificant (*p* < 0.05) reduction (-24.81%) compared to the control group. Compared to the Cd group, the Gin-treated group showed a high statistically-significant amelioration (*p* < 0.01) in plasma level of LH and testosterone with a statistically significant amelioration (*p* < 0.05) in plasma level of FSH, as shown in (Table 1).

2) Tissue levels of MDA, NO, and GSH: We noticed a high statistically significant (p < 0.01) increase in testicular tissue levels of MDA and NO in both the Cd (93.58 and 108.65%, respectively) and Gin-treated (34.86 and 35.34%, respectively) groups compared to the control group. Also, the reduction in tissue levels of MDA and NO in the Gin-treated group was highly statistically significantly (*p* < 0.01) ameliorated compared to the Cd group, as shown in Table 1.

On the other hand, the testicular tissue levels of GSH showed a statistically significant (*p* < 0.05) decrease in Cd and the Gin-treated groups (-60.34% and -19.23%, respectively) compared to rats in the control group. As expected, GSH tissue levels showed statistically significantly (*p* < 0.05) improvement following ginger treatment compared to the Cd group, as demonstrated in Table 1.

### Semen analysis

Semen analysis from the Cd and Gin-treated groups showed a highly statistically significant (*p* < 0.01) reduction in sperm viability (-47.9% and -15.143%, respectively) and motility (-48.47% and -19.51%, respectively) compared to the control group. Moreover, the sperm count showed a highly statistically significant (*p* < 0.01) reduction (-54.53%) in the Cd group and a statistically significant (*p* < 0.05) reduction (-23.98%) in the Gin-treated group compared to the control group. However, the resulting reduction in sperm count, viability and motility seen in the Cd group was highly statistically significantly (*p* < 0.01) improved in the Gin-treated group, as shown in Table 1.

### Electron microscopy (EM)

**FIGURE 1:**
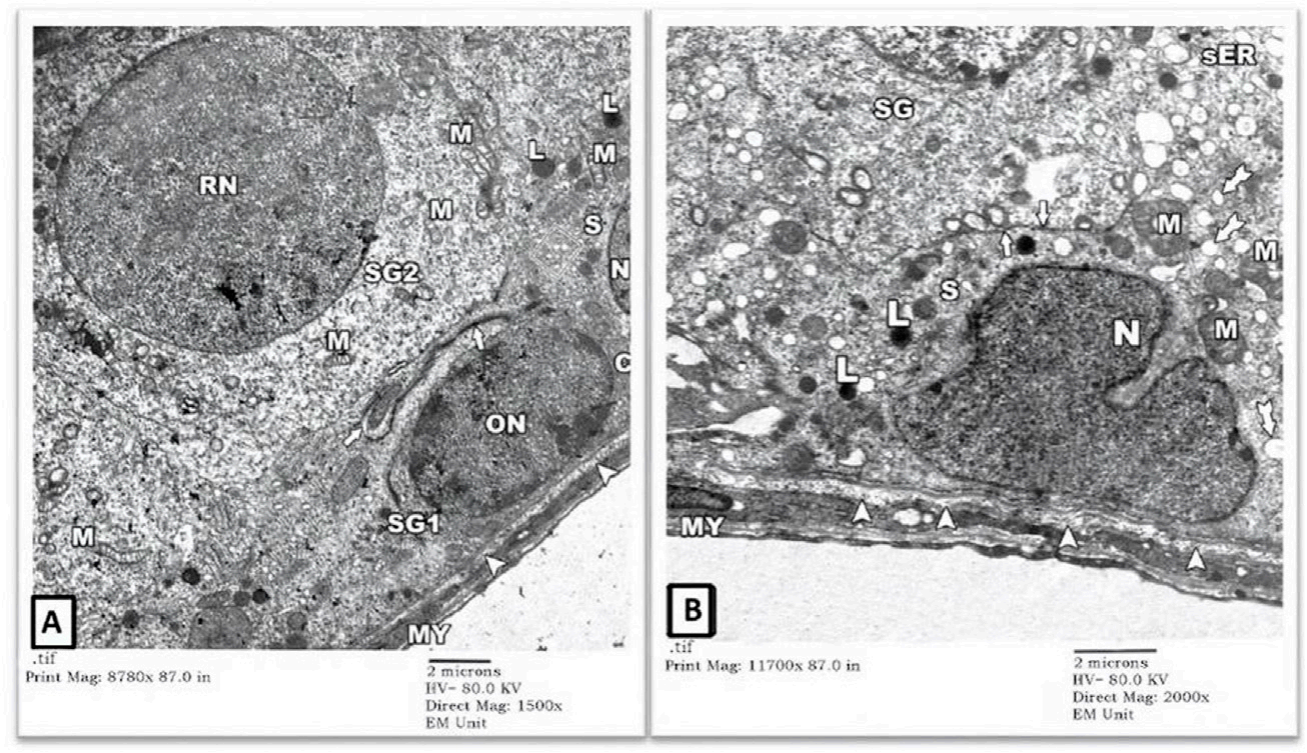
An electron micrograph of a part of seminiferous tubule from testis of a rat in control group showing: **(A)** a spermatogonium (SG1) with an oval nucleus (ON) and a part of Sertoli cell (S) resting on the basement membrane (arrowheads) surrounded by a thin connective tissue sheath containing myoid cells (MY). Sertoli cell exhibits a part of its indented nucleus (N), its irregular cell border (thick arrow), mitochondria (M), numerous vesicles of sER (inside the rectangle), and lysosomes (L). Another spermatogonium (SG2) shows a round nucleus (RN) and mitochondria (M). (Uranyl acetate and lead citrate X 1500, Print Mag. X 8780). **(B)** Sertoli cell (S) resting on the basement membrane shows an indented nucleus (N), mitochondria (M), lysosomes (L), and lipid droplets (tailed arrows). Its irregular cell membrane (thick arrows) separates it from adjacent spermatogonium (SG). (Uranyl acetate and lead citrate X 2000, Print Mag. X 11700).

1) EM Examination of Testes from Control Group: EM examination of ultrathin sections prepared from testes of the control-group rats showed preserved architecture of seminiferous tubules surrounded by a thin connective tissue sheath containing myoid cells (MY). Tubules’ lining cells (Sertoli (S) cells) showed intact indented nuclei (N) with irregular apical (luminal) borders (thick arrow) separating them from adjacent spermatogonia. Their cytoplasm contained many mitochondriae (M), numerous vesicles of the smooth endoplasmic reticulum (sER) of uniform size, lysosomes (L), and lipid droplets (tailed arrows). Normal-looking spermatogonial cells were also seen with intact round (RN)-to-oval (ON) nuclei and several mitochondriae (M) in their cytoplasm. The underlying basal lamina showed well-preserved three layers: lamina Lucida (electron-lucent), lamina densa (electron-dense), and lamina fibro-reticularis (electron-lucent) (Figures 1A,B).

**FIGURE 2:**
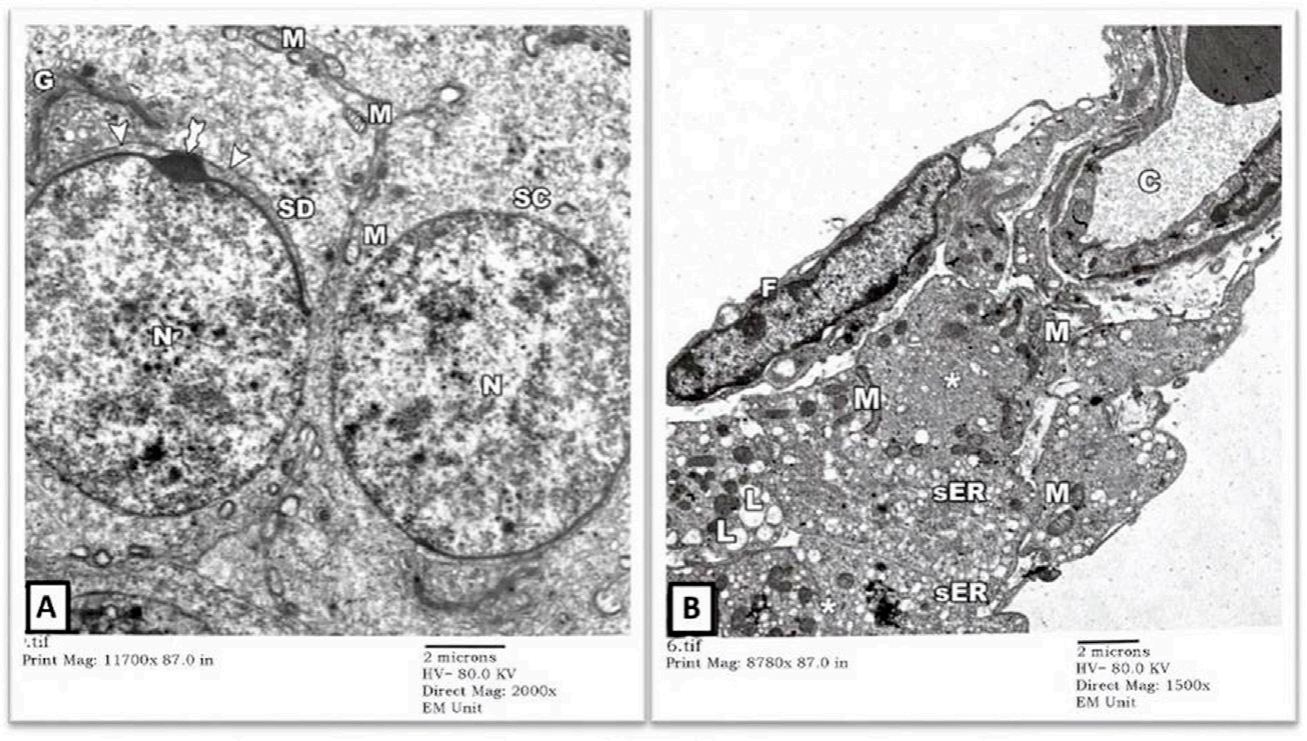
An electron micrograph of the testis from a rat in the control group showing: **(A)** a part of the seminiferous tubule with a spermatocyte (SC) showing rounded nucleus (N) and mitochondria (M). A spermatid (SD) shows a well-developed Golgi apparatus (G). The anterior part of its nucleus is covered by a head cap (arrowheads) and an acrosomal granule (tailed arrow). (Uranyl acetate and lead citrate X 2000, Print Mag. X 11700). **(B)** the testicular interstitium with blood capillaries (C), fibroblasts (F), and Leydig cells (asterisks). The cytoplasm of Leydig cells contains numerous mitochondria (M), vesicles of sER, and lipid droplets (L). (Uranyl acetate and lead citrate X 1500, Print Mag. X 8780).

EM examination also showed normal-looking spermatocytes (SC) with intact cell borders, round nuclei (N), and cytoplasmic mitochondriae (M). Spermatids (SD) showed preserved nuclei with well-developed Golgi bodies (G) and acrosomal vesicles (arrows heads) covering the anterior portions of the nuclei (Figure 2A). The intertubular testicular tissue showed intact capillaries (C), fibroblasts (F), and Leydig cells (asterisks). The cytoplasm of Leydig cells contained numerous mitochondriae (M), sER vesicles, and lipid droplets (L) (Figure 2B).

**FIGURE 3:**
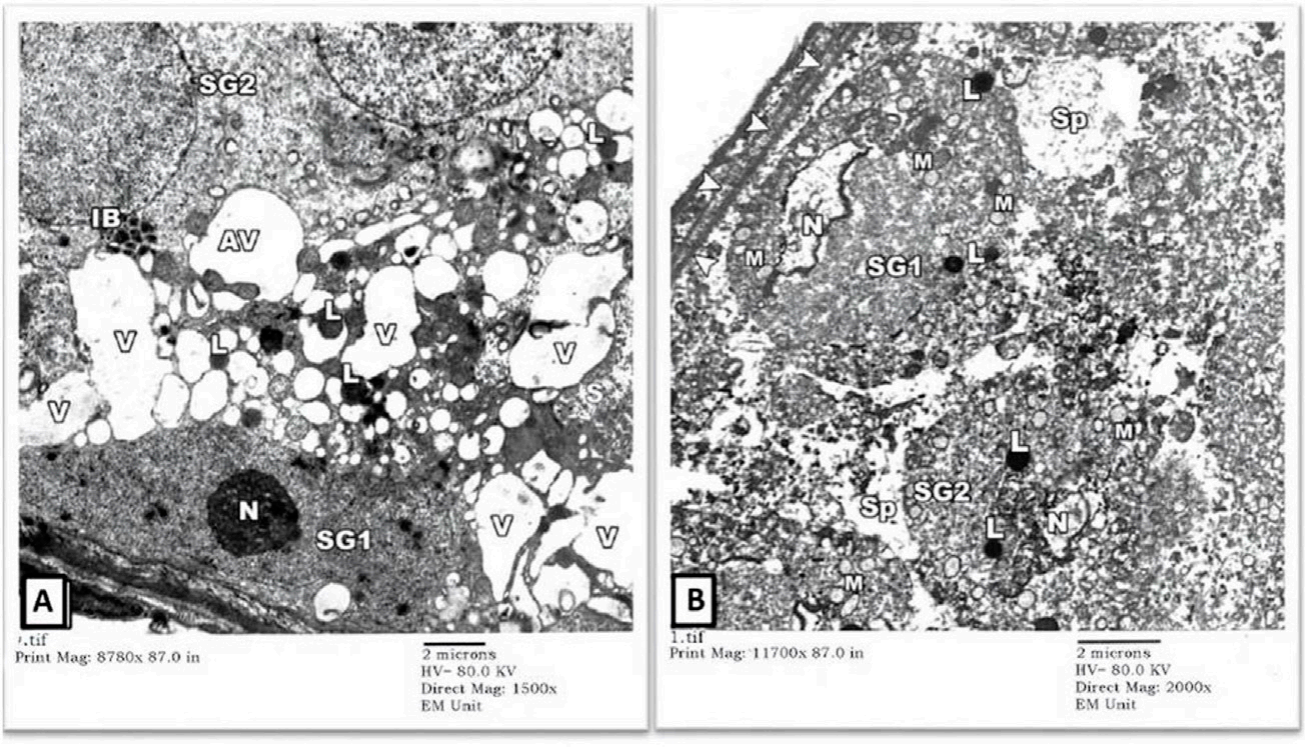
An electron micrograph of a seminiferous tubule of the testis from a rat in cadmium group showing: **(A)** a spermatogonium (SG1) resting on the basement membrane with pyknotic nucleus (N), corrugated nuclear membrane, and dense cytoplasm. The cytoplasm of the Sertoli cell houses variable-sized vacuoles (V) of the distorted sER as well as autophagic vacuoles containing mitochondria (AV) and lysosomes (L). The cytoplasm of another spermatogonium (SG2) contains variable-sized inclusion bodies (IB). (Uranyl acetate and lead citrate X 1500, Print Mag. X 8780). **(B)** a degenerated spermatogonium (SG1) resting on the basement membrane (arrowheads) with nuclear remnants (N), lysosomes (L), and swollen mitochondria lacking cristae (M). Another spermatogonium (SG2) shows an apparent shrinkage with a small distorted nucleus (N) and lysosomes (L). Necrotic debris is seen in the intervening spaces (Sp) between these cells. (Uranyl acetate and lead citrate X 2000, Print Mag. X 11,700).

2) EM Examination of Testes from Cd Group: Sertoli cells of the rats’ testes in the Cd group showed enumerable, variable-sized vacuoles of distorted sER and autophagy vacuoles (AV) containing mitochondria (M) and lysosomes (L). Some spermatogonial cells had pyknotic nuclei (N) with irregular nuclear contours and dense cytoplasm full of variable-sized autophagy vacuoles (AV) (Figure 3A). Some other seminiferous tubules showed degenerated spermatogonial cells (SG1) with nuclear fragmentation (N) (karyorrhexis), swollen mitochondria (M) lacking their cristae and widening of intercellular spaces. Necrotic debris was seen in spaces (Sp) between degenerating spermatogonial cells (Figure 3B).

**FIGURE 4:**
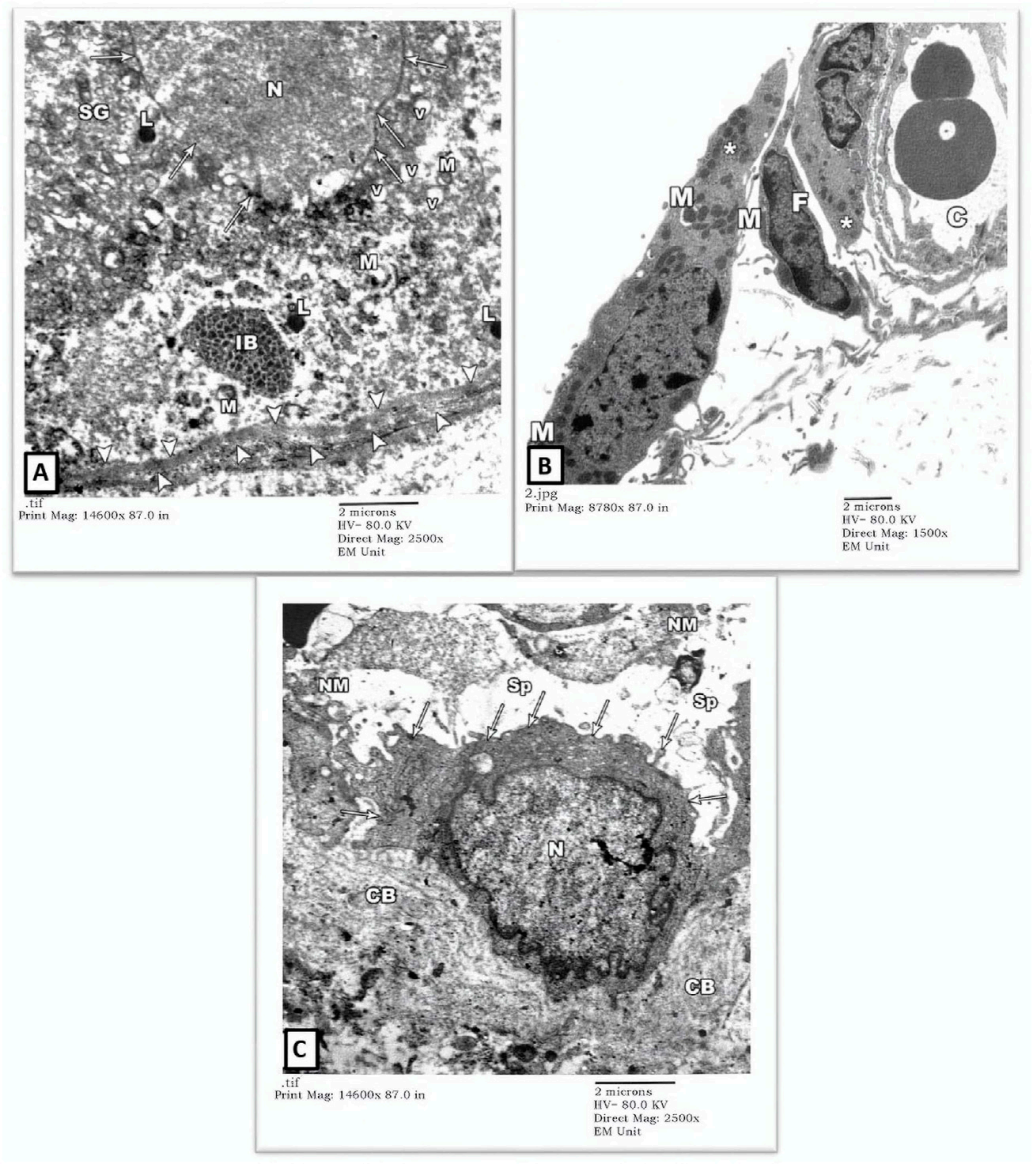
An electron micrograph of the testis from a rat in the cadmium group showing: **(A)** a part of the seminiferous tubule with thick wavy basement membrane (arrowheads). The nuclear membrane of the degenerated spermatogonium (SG) is irregular and disrupted (arrows). The cytoplasm is deranged with vacuoles (V), disrupted mitochondria (M), lysosomes (L), and inclusion bodies (IB). (Uranyl acetate and lead citrate X 2500, Print Mag. X 14,600). **(B)** interstitium with widely spaced interstitial cells and dilated blood capillary (C). The cytoplasm of the Leydig cell (Asterisk) contained some mitochondria (M) but no apparent lipid droplets or sER. Notice the fibroblast (F). (Uranyl acetate and lead citrate X 1500, Print Mag. X 8780). **(C)** interstitium exhibiting a macrophage (arrows) with an irregularly outlined nucleus (N) and collagen bundles (CB) in its vicinity, necrotic material (NM) and wide spaces (Sp). (Uranyl acetate and lead citrate X 2500, Print Mag. X 14,600). interstitium.

The degenerated spermatogonium (SG) showed an irregular, disrupted nuclear membrane (arrows) with deranged cytoplasm containing disrupted mitochondria (M), lysosomes (L), and inclusion bodies (IB). The underlying basal lamina was thicker and more corrugated (arrowheads), with a loss of the normal three-tiered ultrastructure (Figure 4A). Also, there was a remarkable widening of the intertubular spaces (Sp) by extravasated fluids and blood capillary dilatation (C) with subendothelial matrix deposition. The Leydig cells (asterisk) showed nuclear involution with irregular borders and completely lacked cytoplasmic lipid droplets or sER (Figures 4B, C). Extracellularly, collagen bundle (CB) and necrotic material (NM) deposition were noted.

**FIGURE 5:**
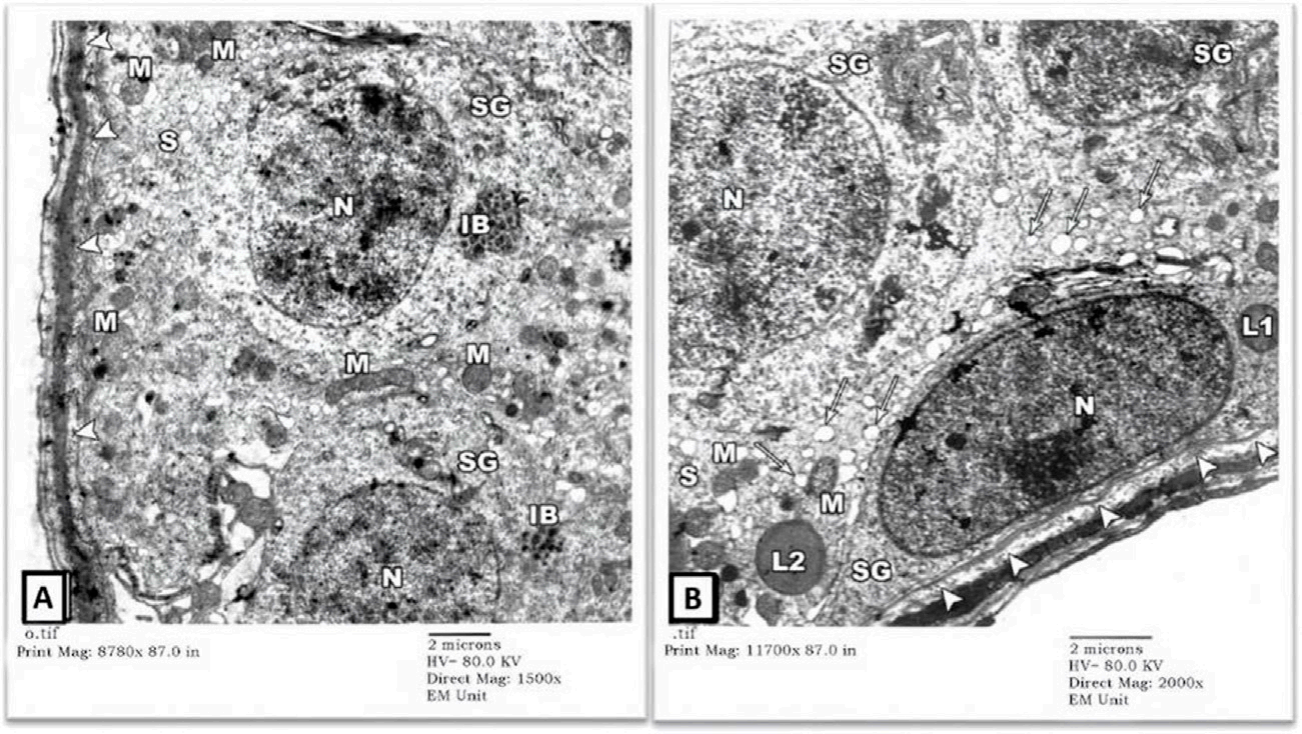
An electron micrograph of the testis from a rat in the Gin-treated group showing: **(A)** a part of the seminiferous tubule exhibiting two spermatogonial (SG) and a part of Sertoli cell (S) resting on a basement membrane (arrowheads) and housing numerous mitochondria (M), and inclusion bodies (IB). All cells, more or less, simulate those of the control rats. (Uranyl acetate and lead citrate X 1500, Print Mag. X 8780). **(B)** a part of seminiferous tubule displaying a spermatogonium (SG) and part of Sertoli cell (S) resting on a thick basement membrane (arrowheads). The cytoplasm of the Sertoli cell shows mitochondria (M), lipid droplets (arrows), and lysosomes (L). These cells regained more or less the control architecture. (Uranyl acetate and lead citrate X 2000, Print Mag. X 11,700).

**FIGURE 6:**
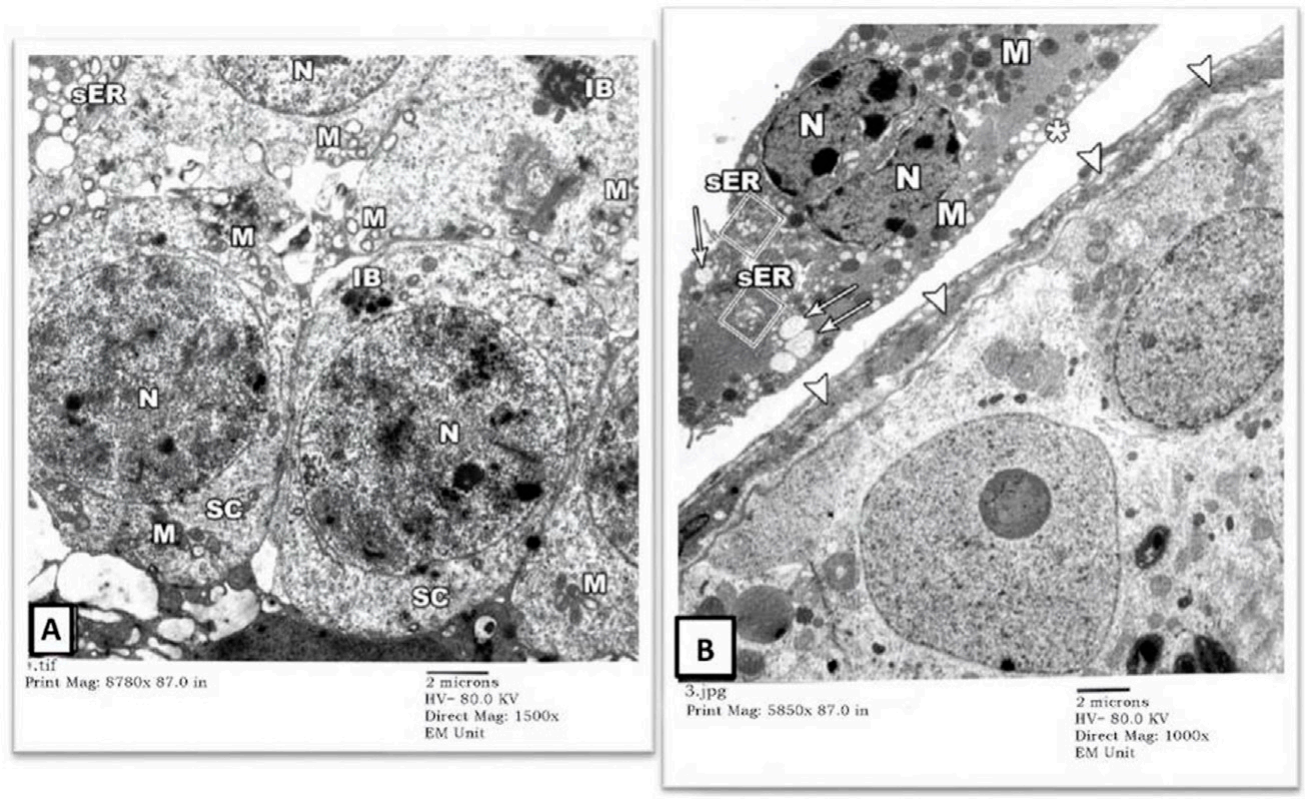
An electron micrograph of the testis from a rat in the Gin-treated group showing: **(A)** a part of seminiferous tubule displaying spermatocytes (SC). Their cytoplasm contains mitochondria (M) and also electron-dense granules (IB). These cells simulate those of the control animals. (Uranyl acetate and lead citrate X 1500, Print Mag. X 8780). **(B)** the interstitium was exhibiting Leydig cell (Asterisk) with vivid nucleus (N), lipid droplets (arrows), mitochondria (M), and sER (inside rectangles). (Uranyl acetate and lead citrate X 1000, Print Mag. X 5850).

3) EM Examination of Testes from Gin-treated Group: Sections from testis of the Gin-treated rats showed largely preserved seminiferous-tubule architecture, with normal-looking spermatogonial (SG) and Sertoli cells (S) resting on the three-tiered basal lamina (arrowheads). Some spermatogonial cells showed blurring of their borders compared to those seen in control-group rats, and few cells showed occasional electron-dense inclusion bodies (IB). Also, there was a mild widening of intercellular spaces (Figures 5A,B). Spermatocytes (SC) showed focal nuclear-chromatin condensation, occasional electron-dense inclusion bodies (IB), and subtle variation in the sizes of sER vesicles (Figure 6A). Leydig cells (asterisk) showed blunting of surface villi, blurring of cell borders, yet preserved lipid droplets (arrows) in their cytoplasm (Figure 6B).

### Light microscopy

**FIGURE 7:**
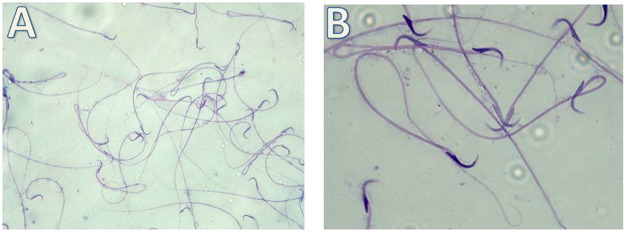
Micrograph illustrating the morphology of sperms of control albino rat Showing normal sperms (H&E; **(A)**-X200, **(B)**-X400)

**FIGURE 8:**
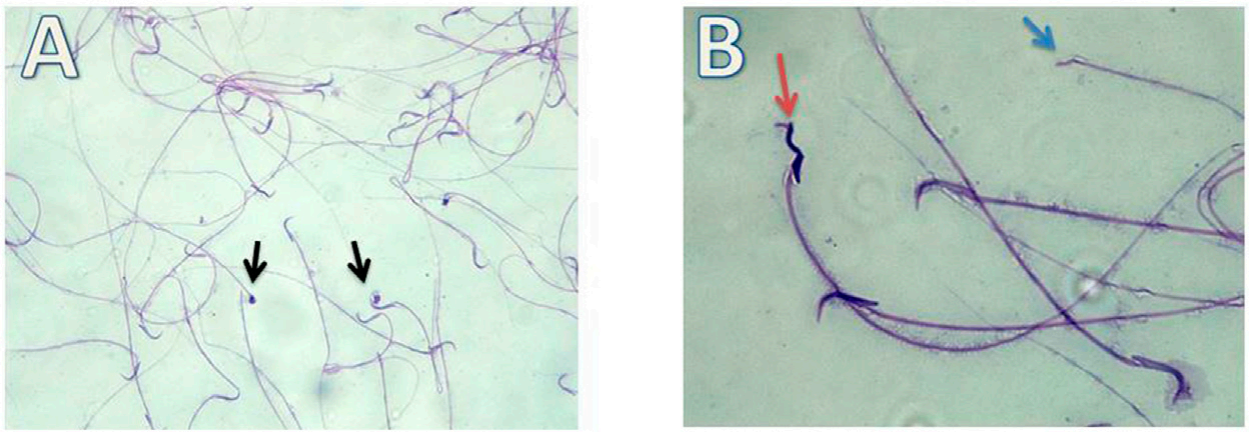
Micrograph illustrating the morphology of sperms of Cd-treated albino rat showing many sperms abnormalities in the form of; absence of head (blue arrow), rounded head (black arrows) and tortuous head (red arrow). (H&E; **(A)**-X200, **(B)**-X400).

**FIGURE 9:**
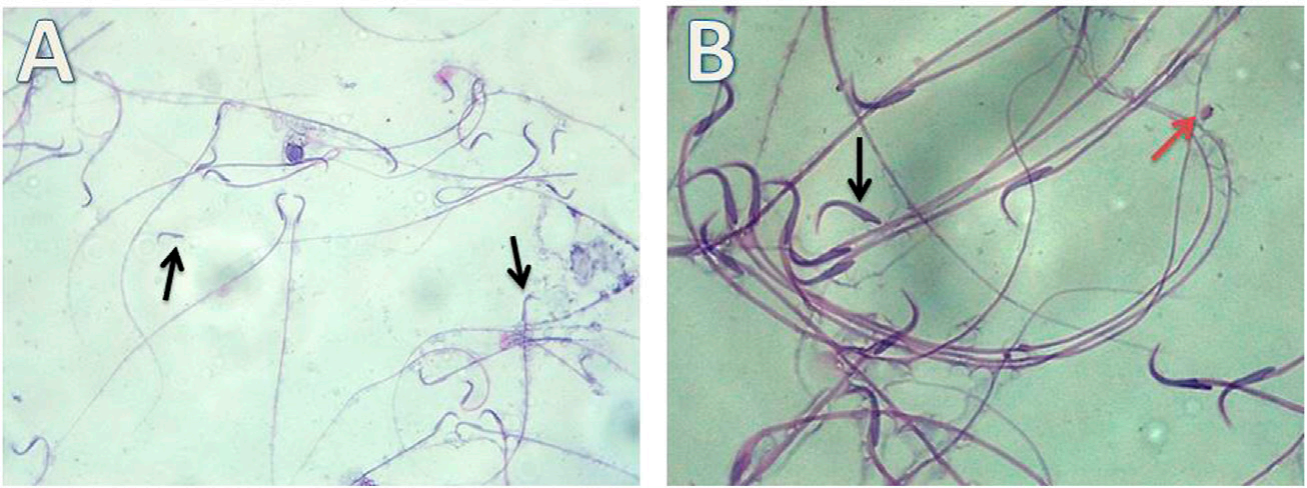
Micrograph illustrating the morphology of sperms of Gin-treated albino rat showing some sperms abnormalities as detached head (black arrow)and rounded head (red arrow) (H&E; **(A)**-X200, **(B)**-X400).

**FIGURE 10:**
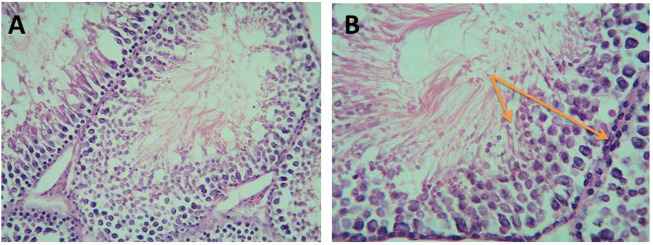
Micrograph of control albino rat testes showing normal seminiferous tubule lined with thick germinal epithelium containing different stages of spermatogenic cells (orange arrows) and supportive Sertoli cells with many sperms in the lumen. The interstitial connective tissue contains Leydig cells and fibroblasts (H&E; **(A)**-X200,** (B)**-X400).

i. Detection of morphological changes of the spermatozoa:

Our results showed highly statistically significant (*p* < 0.01) sperms abnormalities in the Cd (absent, rounded and tortuous head) and the Gin-treated (detached and rounded head) groups compared to the control group. Meanwhile, the sperms abnormalities were highly statistically significantly (*p* < 0.01) ameliorated in the Gin-treated group compared to the Cd group, as shown in Table 1; Figures 7–9.

ii. Detection of histological changes of the testis:

As shown in Figure 10, histological examination of the testicular sections of the control group revealed normal seminiferous tubules. They were lined by a stratified layer of the germinal epithelium at different stages of spermatogenesis (from primary spermatocytes at the basement membrane to sperms at the lumen) with Sertoli cells in between. The flagella of mature sperms were seen filling the lumina of the seminiferous tubules. The interstitial spaces in-between the tubules contained Leydig cells with some fibroblasts.

**FIGURE 11:**
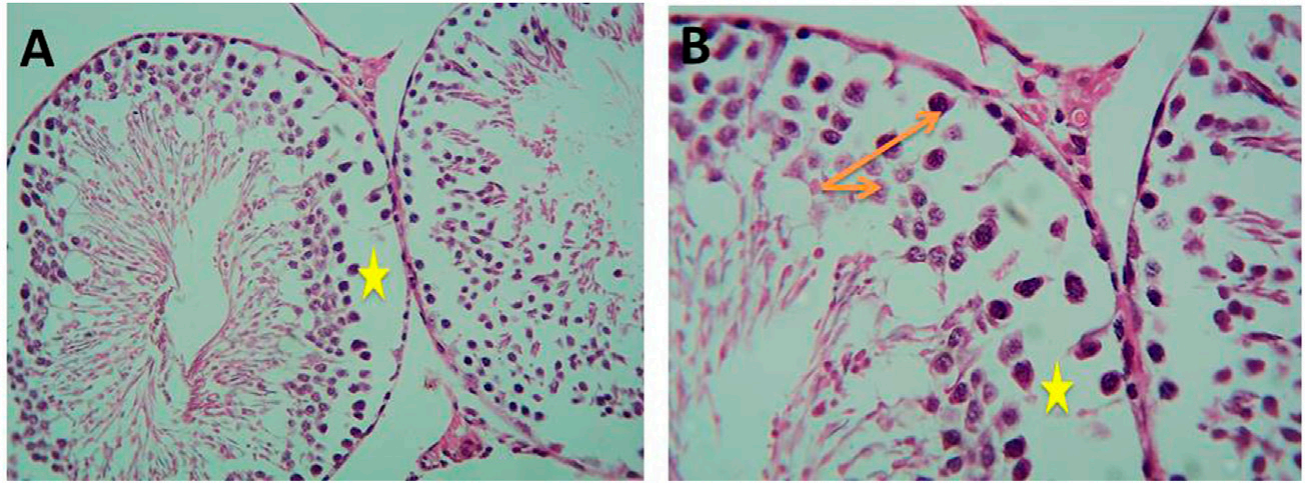
Micrograph of Cd-treated albino rats showing decreased thickness of germ cell layer (orange arrows) and detached cells with wide spaces between them (yellow stars) (H&E; **(A)**-X200, **(B)**-X400).

**FIGURE 12:**
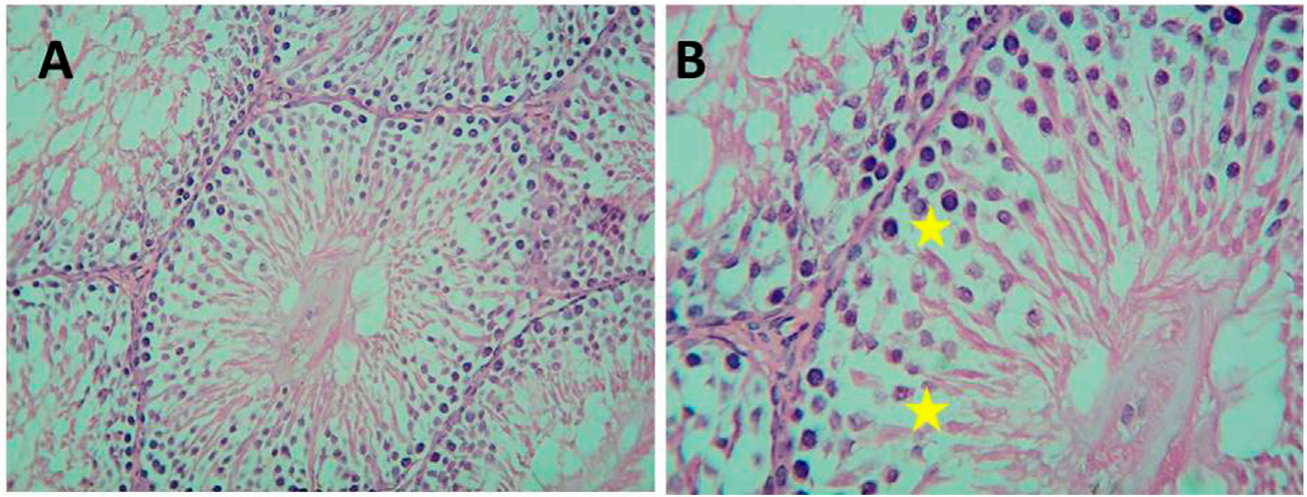
Micrograph of Gin-treated albino rat showing less crowded spermatogenic cells than normal control with some spaces between the cells (H&E; **(A)**-X200,**(B)** B-X400).

Histological examination of testicular sections of Cd-treated rats revealed impaired spermatogenesis and detached cells with a decreased thickness of the germ cell layer, but focal spermatogenesis was noticed in the sections in the form of some spermatids. On the other hand, histological examination of testicular sections of Gin-treated rats revealed a slight decrease in the spermatogenic cells with small spaces in between, as demonstrated in Figures 11, 12.

## Discussion

Heavy metals are chemical elements of high density and toxic effects on human tissues, even at low concentrations (Chandra *et al.,* 2017). Bio-accumulation and subsequent intoxication occur as heavy metals are usually taken up by (or precipitate in) tissues faster than elimination or excretion (Shahid *et al.,* 2021). A higher rate of heavy metals deposition is particularly noticed in the rapidly dividing cells of the gastrointestinal, hematopoietic, reproductive, and central nervous systems (Pizzaia *et al.,* 2019; Zhou *et al.,* 2020).

Exposure to Cd primarily occurs through ingesting contaminated food and water and, to a significant extent, through inhalation and cigarette smoking. It accumulates in plants and animals with a long half-life of about 25–30 years (Genchi *et al.,* 2020). Long-term exposure to Cd, with subsequent organ/tissue buildup, leads eventually to multiorgan failure, e.g., chronic renal dysfunction progressing to failure (Chen *et al.,* 2016), chronic obstructive pulmonary disease (Hutchinson *et al.,* 2018), osteomalacia, osteoporosis, and even cancers (e.g., lung and breast cancers) (Filippini *et al.,* 2020; Li *et al.,* 2020). Some researchers found a correlation between Cd/lead exposure and increased blood pressure and myocardial contractility changes in animals, although supporting data in humans are lacking (Protsenko *et al.,* 2020).

Several animal studies reported the toxic effects of Cd on the reproductive system (Heidari Khoei *et al.,* 2019). In male rats, testicular dysfunction was attributed to a direct effect on hormone-secreting Leydig cells, possibly secondary to dysfunction of the hormone-regulating hypothalamic-hypophyseal-testicular axis. Although earlier studies suggested that Cd deposition in the hypothalamus or pituitary gland is the mechanism for inducing testicular hypofunction (Rinaldi *et al.,* 2017); other studies showed that infertility is caused primarily by testicular dysfunction as Cd-induced toxic changes in testicular tissue seen in various animal models at different stages of maturation (de Angelis *et al.,* 2017; Drąg-Kozak *et al.,* 2018).

The current study showed that rats exposed to Cd through ingestion had decreased testicular functions in the form of reduced testicular weight and decreased plasma testosterone, FSH and LH levels. Our study also showed increased degenerated spermatogonial cells, autophagy vacuoles, and significant nuclear and cytoplasmic changes in Cd-exposed rats that were remarkably reduced after ginger administration. Our findings are supported by previously published data investigating the protective effect of ginger against testicular tissue damage caused by various toxins (Fekry *et al.,* 2019; Ali *et al.,* 2020; Fang *et al.,* 2020).

Our study demonstrated decreased testosterone, LH, and FSH levels in the Cd-exposed rats, findings that agree with previously published data on the effect of CdCl_2_ on the pituitary-gonadal axis by Nna *et al.* (2017), Wang *et al.* (2021), and Horri *et al.* (2021). The reduced FSH levels could be explained by Cd-induced apoptosis in the anterior pituitary cells in a dose-dependent manner (Yang *et al.*, 2005). Concerning LH and steroidogenesis, CdCl_2_ injection was reported to significantly increase NO production (Waisberg *et al.*, 2003) and decrease testosterone secretion with a direct impact on the pituitary gland and the resulting LH synthesis inhibition (Dobashi *et al.*, 2001).

Testosterone production enhancement is still the eventual goal for many scientists due to the critical testosterone function as the major sex hormone in males (Kelly and Jones, 2013). Testosterone plays a vital role in promoting the development of male sex organs and other sexual characteristics such as body hair growth and increased bone and muscle mass (Mooradian *et al.*, 1987). Testosterone deficiency is linked to several diseases in males as infertility, diabetes, osteoporosis, and bone loss (El-Migdadi *et al.*, 2005; Yarrow *et al.*, 2017; Karakas *et al.*, 2018). Therefore, many studies investigated the effect of dietary supplements and medicinal plants on testosterone level.

Several natural herbal compounds were investigated for their protective and possible therapeutic effects against Cd-induced tissue damage (Liu L. et al., 2015; Rafiee *et al.,* 2019). Ginger extract has potent anti-inflammatory, antioxidant (Arablou *et al.,* 2014), and anti-diabetic properties (Hester *et al.,* 2018) comparable to pharmacological agents (Marettová *et al.,* 2015). Our study showed a significant protective effect of ginger administration on testicular functions as evidenced by reduced testicular-weight loss and higher plasma testosterone, FSH and LH levels compared to Cd-exposed rats.

Previous mice-model studies postulated several possible mechanisms to explain the pathogenesis of Cd-induced testicular dysfunction and infertility, such as (a) overexpression of apoptotic pathway proteins through overexpression of cytokines, leading to testicular cell necrosis and defective steroidogenesis and spermatogenesis (Ogawa *et al.,* 2013; Ghafoor *et al.,* 2020); (b) oxidative stress leading to hemorrhagic necrosis and cellular degeneration (Oyewopo and Olaniyi, 2017); (c) downregulation of nitric oxide synthesis with subsequent endothelial cell dysfunction, microvasculature defect, and blood-testis-barrier disruption (Bashandy *et al.,* 2016); and (d) Cadmium-calcium (Cd–Ca^+2^) interaction that competes with calmodulin for Ca^+2^, blocking the downstream calmodulin-dependent signaling pathway (Ren *et al.,* 2019).

The cytotoxic effects induced by oxidative stress on different body tissues are well known (Sakr *et al.,* 2016a), so as the counter-protective effects of natural and herbal compounds (Sakr *et al.,* 2016b). ROS accumulation in tissues induces chronic inflammation and fibrous tissue formation (Aprioku, 2013). Our results support the oxidative damage theory. We found increased testicular tissue levels of MDA reflecting lipid peroxidation, with reduced levels of the free radical-scavenger (GSH), denoting increased cellular oxidative stress in the Cd-exposed rats. The results also agree with the increased MDA levels that Gabr *et al*. (2019) found in their work.

The rich antioxidant properties of ginger contribute to its free radical scavenging capacity (Sultana *et al.,* 2010). Ginger also modulates phase II detoxification enzyme, reduced glutathione, and biochemical pathways activated in chronic inflammation, explaining its chemopreventive and antioxidant potentials and its protection of biological tissues and cell membrane lipids (Wu *et al.,* 2008; Suresh *et al.,* 2010). The phenolic phytochemical constituents of ginger, like zingerone and [6]-Gingerol of ginger, can suppress the chain reactions of lipid peroxidation, scavenge various free radicals such as superoxide and peroxyl radicals, and inhibit the generation of inducible nitric oxide synthase (iNOS) and the production of nitric oxide (NO) in all biological systems (Gabr *et al.,* 2019). The vitamin content of ginger, especially vitamins A, B6 and retinoids, plays a vital role in body growth, fat reserves, and protein synthesis (Ali *et al.,* 2008). All these effects contribute to the kinetics of chelation of heavy metals (Haleagrahara *et al.,* 2010) and their elimination from various body tissues.

## Summary and conclusion

The toxicity of Cd is associated with several deleterious effects on different body organs/tissues, including testes. Exposure to Cd resulted in local testicular ROS generation and pituitary-induced toxic effects manifested as decreased testicular weight and decreased plasma testosterone, FSH and LH levels with increased degenerated spermatogonial cells and autophagy vacuoles, and significant nuclear and cytoplasmic changes. Ginger administration could oppose Cd-induced testicular changes through its rich phenolic phytochemical and vitamin content that has free radical scavenging, anti-inflammatory, antioxidant and anti-lipid peroxidation effects.

## Data Availability

The original contributions presented in the study are included in the article/supplementary material. Further inquiries can be directed to the corresponding author.

## Abbreviations

cadmium; LH: luteinizing hormone; FSH: follicle-stimulating hormone; EM: electron microscope; MDA: malondialdehyde; NO: nitric oxide; GSH: reduced glutathione; sER: smooth endoplasmic reticulum; TBA: thiobarbituric acid; ROS: reactive oxygen species; NOS: nitric oxide synthase.
